# Supplementary material for: Bivariate genome-wide association meta-analysis of pediatric musculoskeletal traits reveals pleiotropic effects at the SREBF1/TOM1L2 locus
Source: Nat Commun. 2017 Jul 25;8:121. doi: 10.1038/s41467-017-00108-3 (PMC5527106; doi:10.1038/s41467-017-00108-3)
Supplement: Supplementary file 5 — Supplementary Information [file 41467_2017_108_MOESM5_ESM.pdf]

File name: Supplementary Information
Description: Supplementary Figures, Tables, Notes and Method.

File name: Supplementary Data 1
Description: Genome-wide significant SNPs for the bivariate meta-analysis of total body bone mineral density (TBLH-BMD). Estimates were derived from 10,414 children participants of 4 different pediatric studies worldwide. Beta coefficients are reported for the A1 allele.

File name: Supplementary Data 2
Description: Genome-wide significant SNPs for the bivariate meta-analysis of total body bone mineral density (TBLH-BMD) and total body lean mass (TB-LM). Estimates were derived from 10,414 children participants of 4 different pediatric studies. Beta

File name: Supplementary Data 3
Description: Association of markers in the 17p11.2 loci with previous GWAS in musculoskeletal traits. All effect sizes are reported for the allele 1 (A1). TB-LM = Total body Lean Mass assessed in Zillikens et al. [27] FN-BMD= femoral neck bone mineral density and LS-BMD=lumbar spine BMD, assessed in Estrada et al. [20]. Highlighted in red are the markers considered to exert pleiotropic effects in both traits

File name: Supplementary Data 4
Description: Blood cis-eQTL (FDR<0.05) for TBLH-BMD and TB-LM bivariately associated SNPs and proxies in 17p11.2. Quantities under the gene headers represent z-scores for the cis-expression effect of allele A1 on that particular gene. Positive values denote an increase in expression and negative values a decrease in gene expression. SNPs present in the analysis are shadowed. Proxies are chosen to be in high LD (r2>0.8) with analyzed SNPs (GWS). Effect sizes and P-values for the univariate and bivariate associations are also provided. Missense variants are in bold font.

File name: Peer Review File
Description:

Supplementary Figures

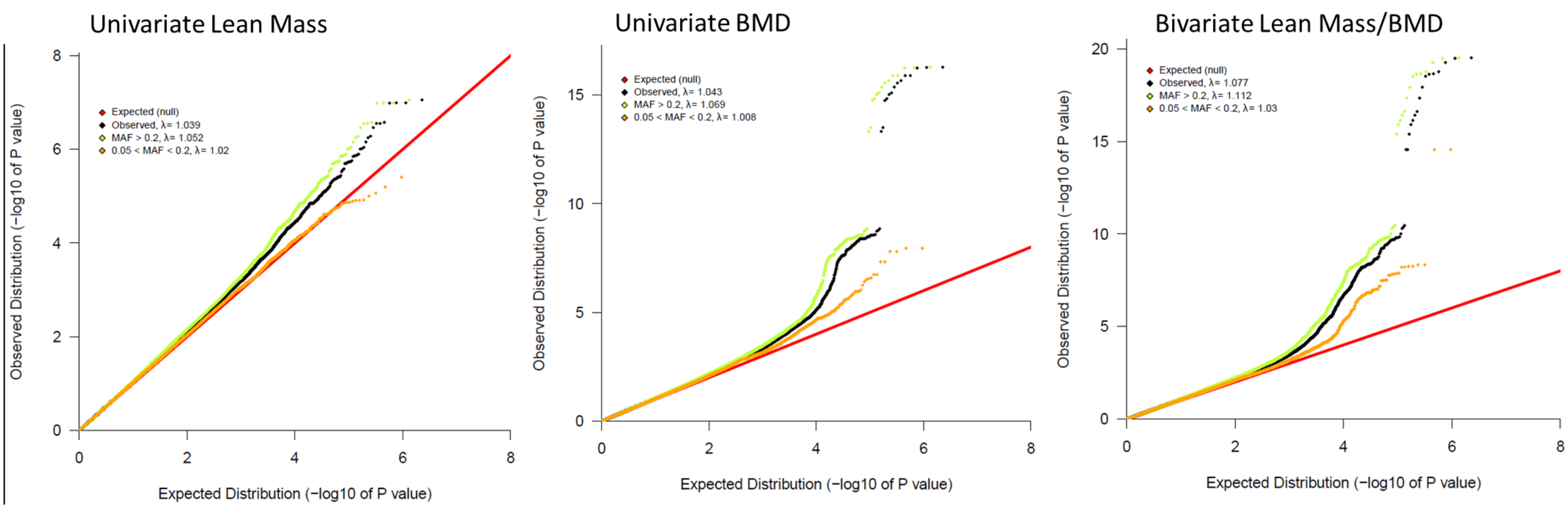

Supplementary Figure 1. QQPlots for the genome-wide meta-analyses. Quantile-Quantile plots showing P-Values for association, deviating from the null hypothesis of no association (identity line), per minor allele frequency category.

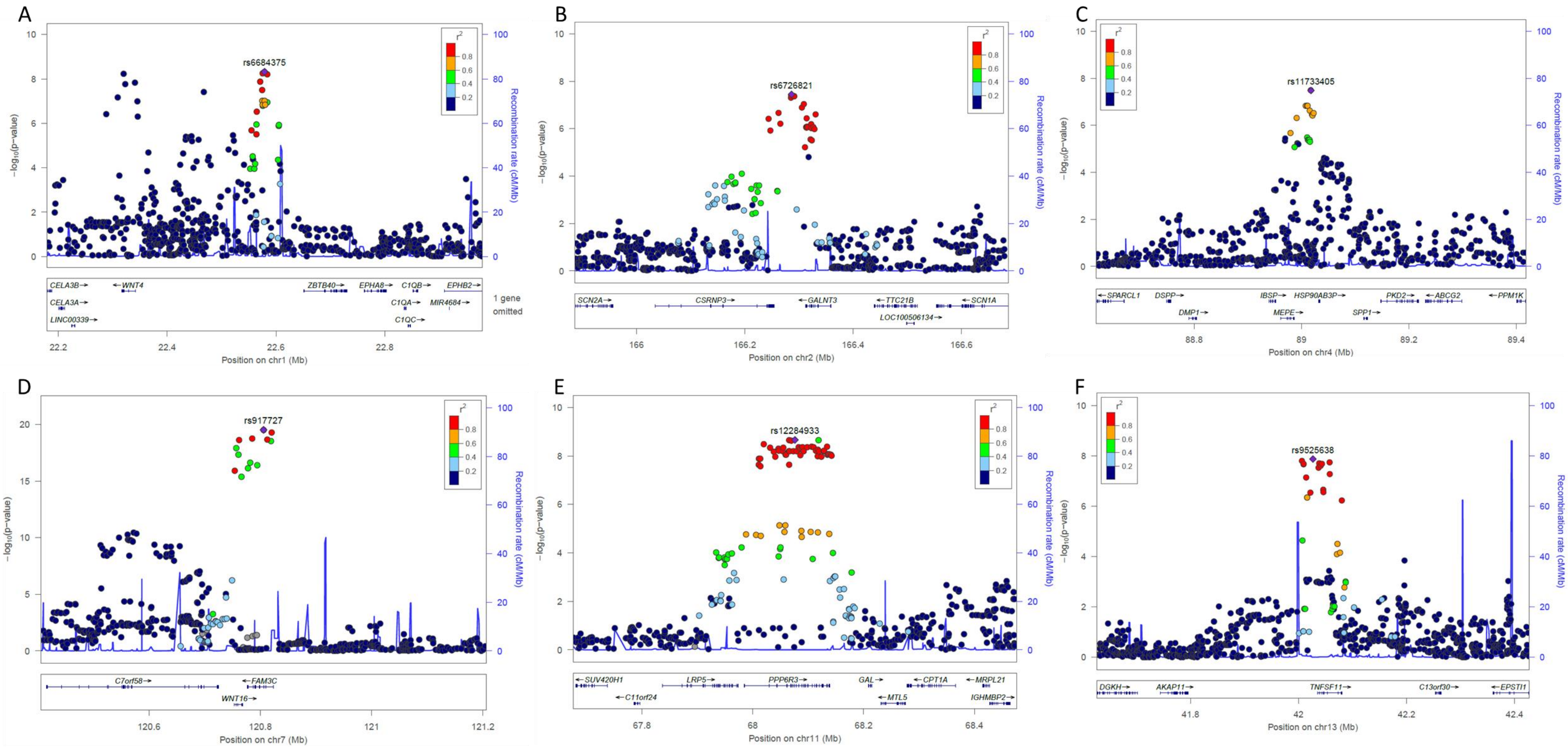

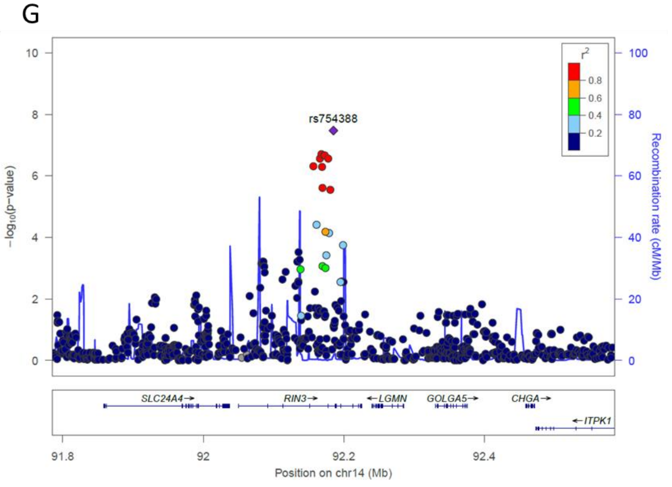

Supplementary Figure 2. Regional plots. Regional association plot for each of the genome-wide significant loci. (A. 1p36.12, B. 2q24.3, C. 4q22.1, D. 7q31.31, E. 11q13.2, F. 13q14.11, G.14q.2.12). SNPs are plotted by position in a 1Mb window against association with the bivariate LM/BMD ($-\log_{10}$ P). Plot highlighting the most significant SNP in the meta-analysis . Blue peaks indicate recombination rates. The SNPs surrounding the most significant SNP are color coded to reflect their LD with this SNP (from pairwise r^2 values from the HapMap CEU). Genes, exons and the direction of transcription from the UCSC genome browser are noted.

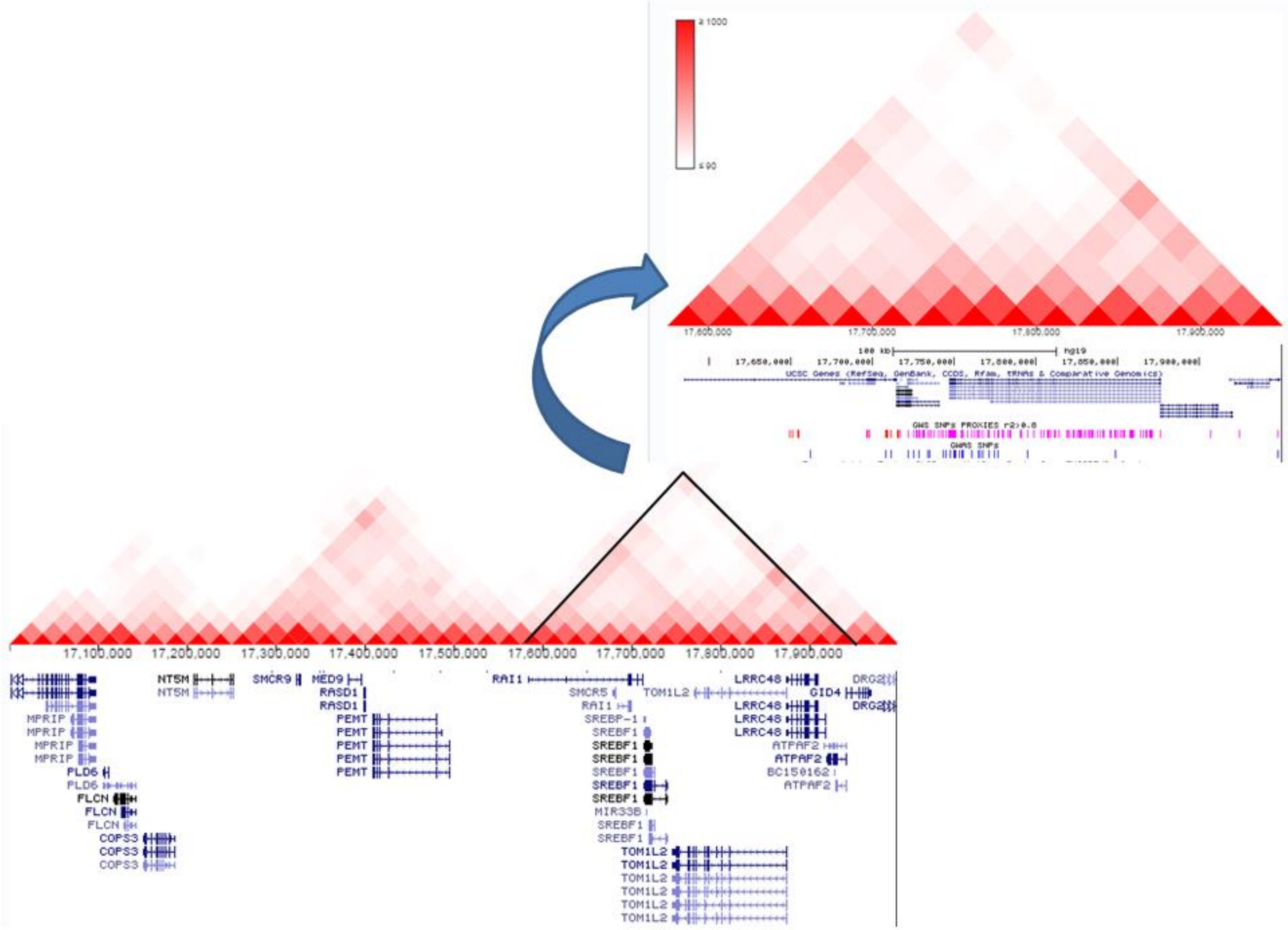

Supplementary Figure 3. A topological associated domain (TAD) includes associated variants from *RAI1, SREBF1, TOM1L2*, *LRRC8* and *ATPAF2*. In line with the chromatin interactions, TADs in K562 cell line suggest a complex regulation structure in the 17p11.2 locus.

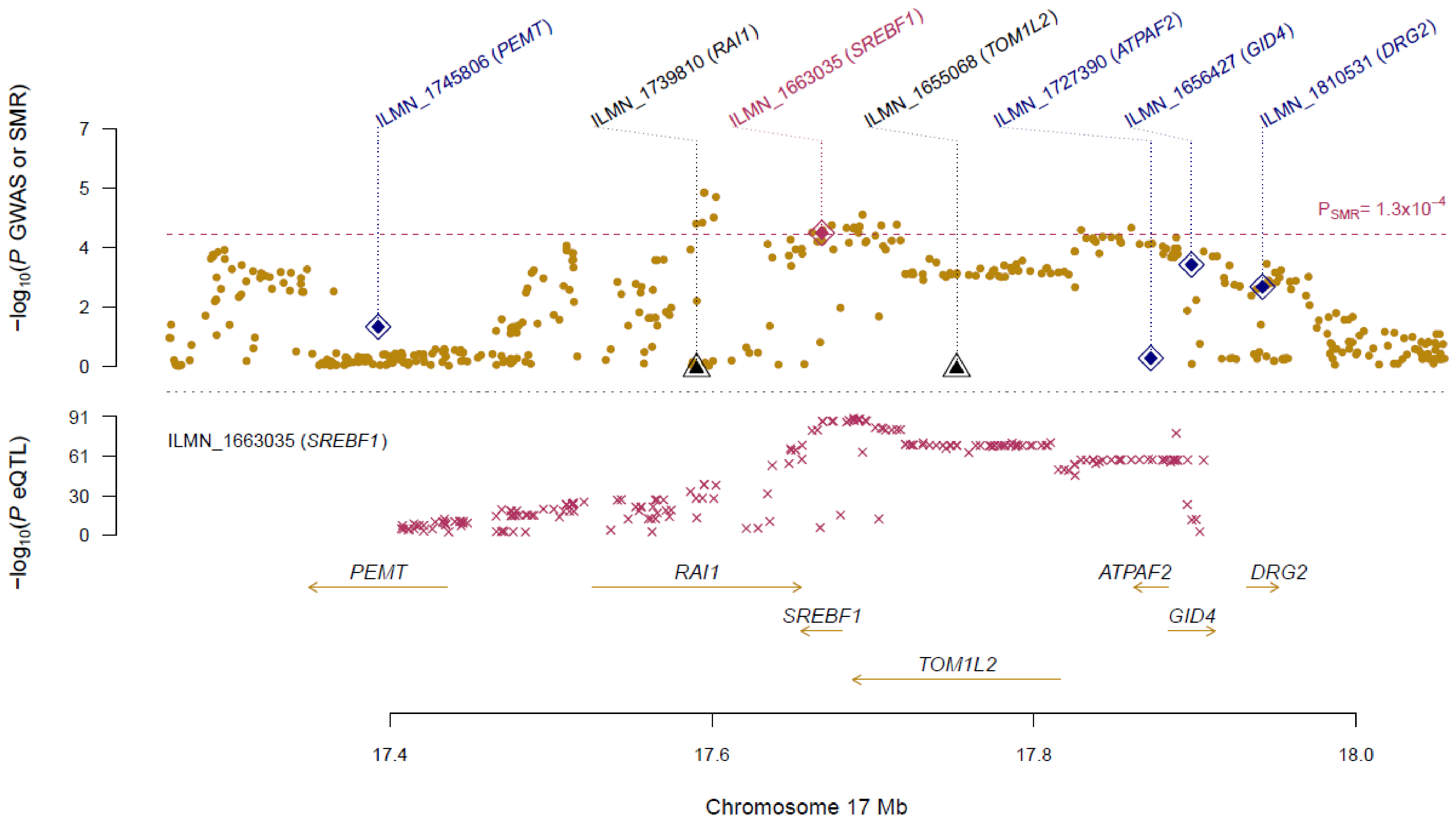

Supplementary Figure 4. Prioritization of genes at the 17p11.2 using SMR analysis. Top plot, Brown dots represent the Pvalues for SNPs in our lean mass GWAS meta-analysis, diamonds represent the P values for probes from the SRM test and triangles represent probes without a cis-eQTL at $P_{eQTL} < 5 \times 10^{-8}$. **Bottom plot**, the eQTL P-values of SNPs from the Westra et al.[1] study for the ILMN_1663035 probe tagging *SREBF1.* The top and bottom plots include all the SNPs available in the region in the GWAS and eQTL summary data, respectively, rather than only the SNPs common to both data sets. Highlighted in red is the probe tagging *SREBF1* that passed the SMR and HEIDI tests.

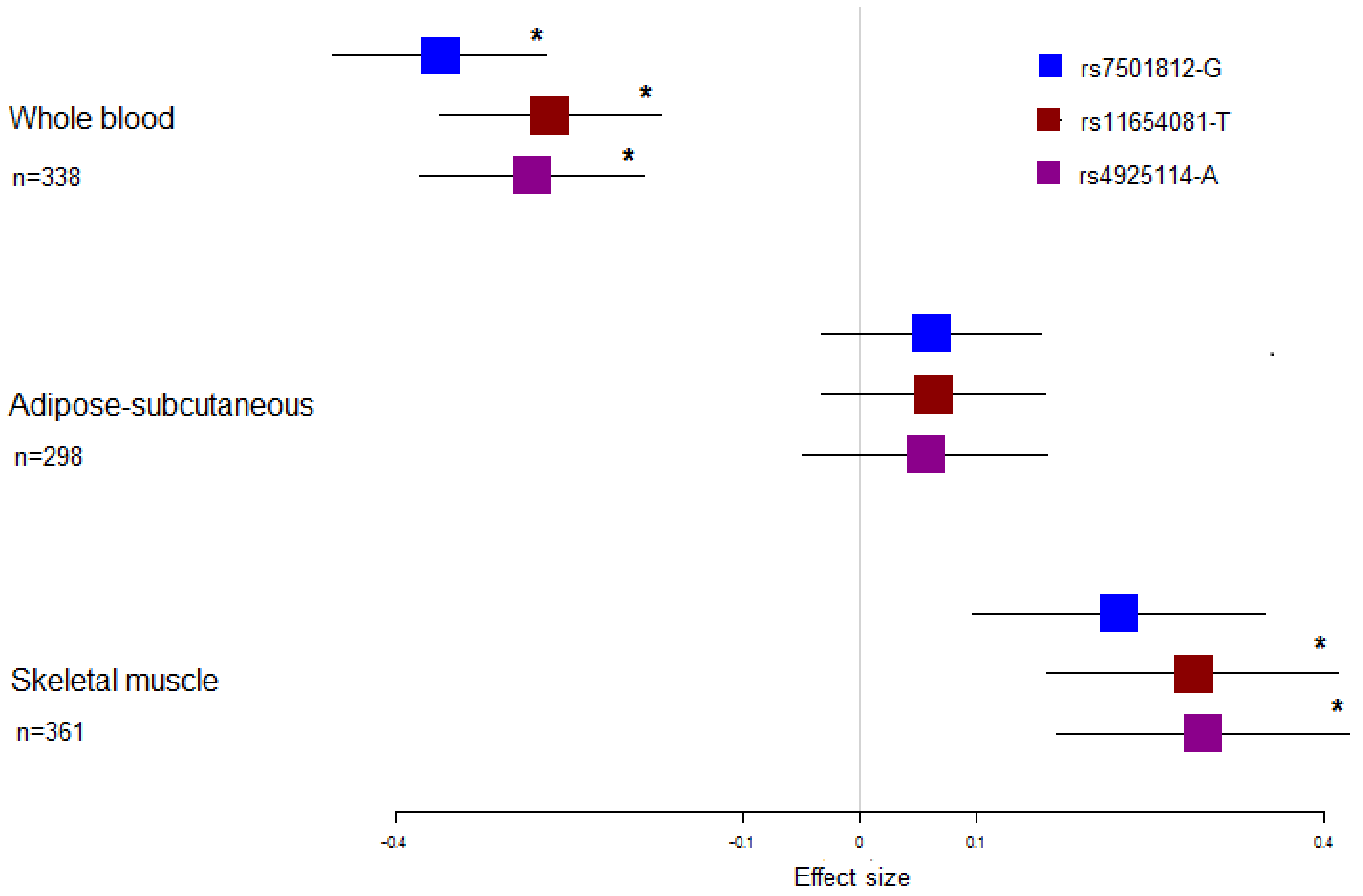

Supplementary Figure 5. Multi-tissue *SREBF1* eQTL comparison based on GTEx data[2]. The effect size and s.d. of the Whole blood (338 samples), Adipose-subcutaneous (298 samples) and Skeletal muscle (361 samples) eQTLs is defined as the slope of normalized expression versus the three genotypes. All analyses are adjusted for sex, genotyping array platform, PEER factors and genetic principal components. Effects showing an FDR<0.05 are marked with an asterisk.

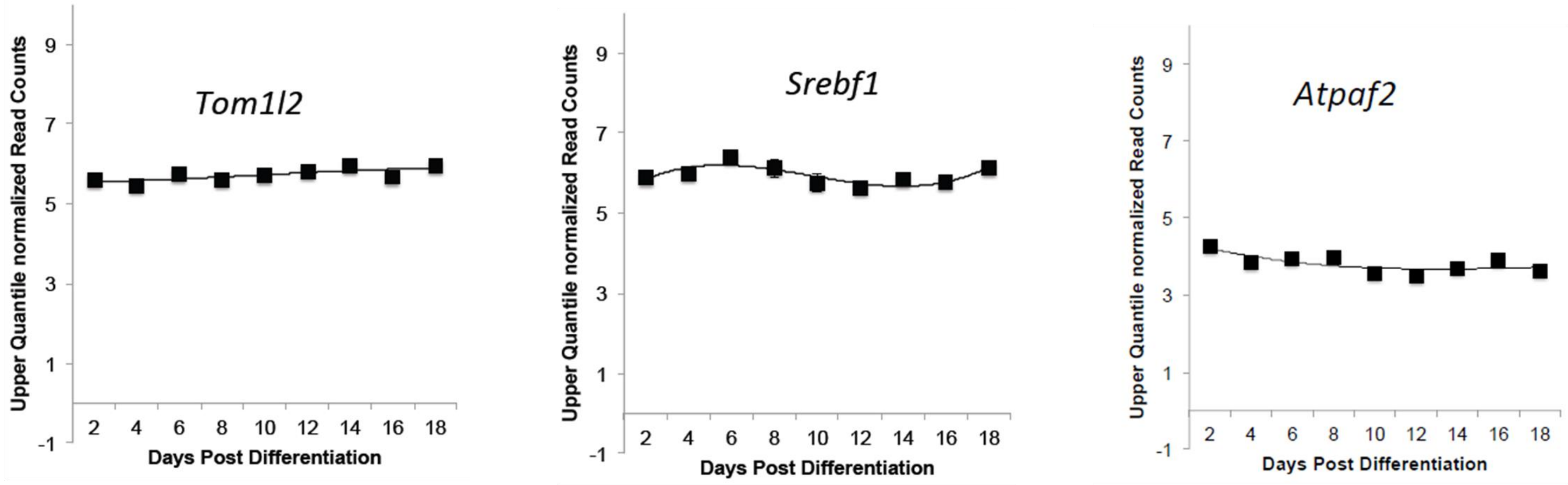

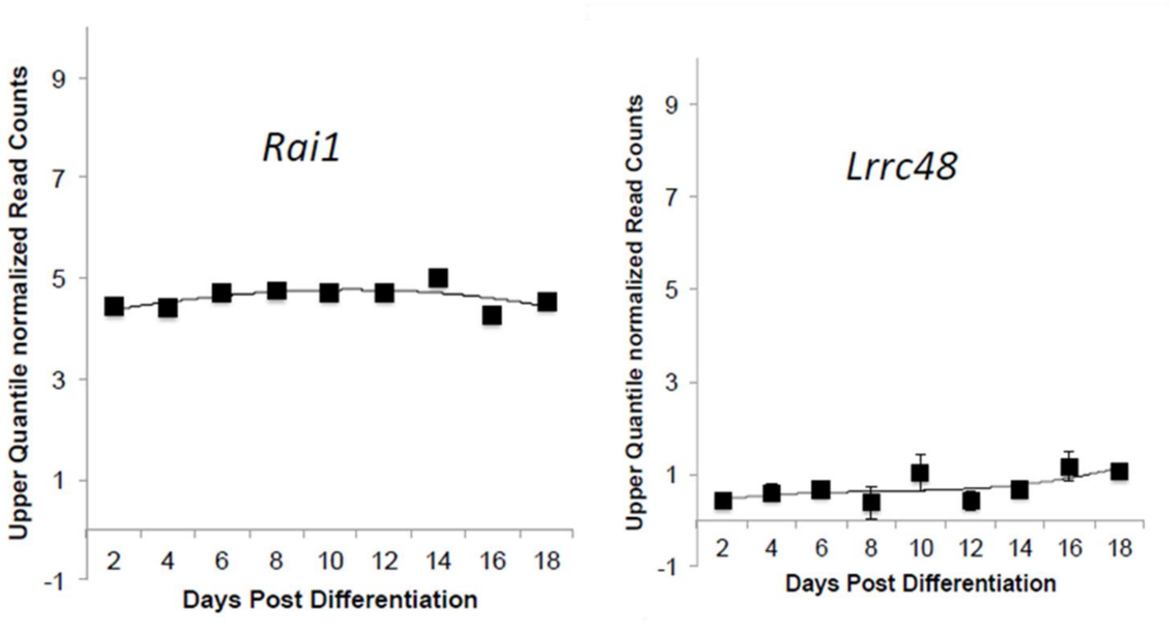

Supplementary Figure 6. Gene expression profiles of *Tom1l2, Srebf1, Atpaf2, Rai1* and *Lrcc48* measured throughout the osteoblast maturation process. Measurements were performed in cells extracted from mouse calvariae, as measured by RNAseq. Samples for expression purposes were collected every other day for 18 days, starting 2 days after the cells were first exposed to an osteoblast differentiation cocktail. Relative transcript abundance is expressed as the number of query transcripts per million unique transcripts (transcripts per million), after normalizing to the upper quartile. A local weighted scatterplot smoothing curve was plotted to help with visualizing the expression pattern.

Supplementary Tables

Supplementary Table 1. Genes Located within the 17p11.2 locus. We here summarize the information available on the genes located in 17p11.2 related to musculoskeletal traits. OMIM annotations are only considered if they present with a musculoskeletal phenotype. Expression in murine osteoblast are based in clavaria data (see Methods). Expression in skeletal muscle is based in GTEx data[2-4]

Gene Symbol	Gene name and Known function	OMIM annotation	Mice skeletal phenotype	Expression in murine osteoblast	Expression in Skeletal muscle
RAI1	Retinoic acid-inducible 1. Although the function of this protein is unknown, it is thought to be involved in nervous system development.	Smith-Magenis Syndrome	Knockout mice with malformations in both the craniofacial and the axial skeleton	yes	+
TOM1L2	Target of myb1-like 2. Probable role in protein transport. May regulate growth factor-induced mitogenic signaling.	no	Kyphosis	yes	+++
C17orf39	Also known as *GID4* (GID complex subunit 4 homolog). The protein encoded by this gene is thought to be a subunit of the Mediator complex, which is required for activation of RNA polymerase II transcription by DNA bound transcription factors.	no	no KO available	yes	++
ATPAF2	ATP synthase mitochondrial F1 complex assembly factor 2. This gene encodes an assembly factor for the F(1) component of the mitochondrial ATP synthase. This protein binds specifically to the F1 alpha subunit and is thought to prevent this subunit from forming nonproductive homooligomers during enzyme assembly.	no	Decreased number of thoracic vertebrae, abnormal maxilla and shape of vertebrae, and low body weight	yes	+
SREBF1	Sterol regulatory element binding transcription factor 1. This gene encodes a transcription factor that binds to the sterol regulatory element-1 (SRE1), which is a decamer flanking the low density lipoprotein receptor gene and some genes involved in sterol biosynthesis.	no	no information about skeletal phenotype has been reported for existing transgenic models.	yes	++
LRRC48	Also known as *DRC3* , Dynein Regulatory Complex Subunit 3.	no	no KO available	no	0
MYO15A	Myosin XVA, encodes an unconventional myosin. Studies in mice suggest that it protein is necessary for actin organization in the hair cells of the cochlea. Mutations in this gene have been associated with profound, congenital, neurosensory, nonsyndromal deafness.	no	Increased Grip strength, high body fat amount	no	0
DRG2	Developmentally regulated GTP binding protein 2. This gene encodes a GTP-binding protein known to function in the regulation of cell growth and differentiation.	no	Decreased Body Weight, longer tibia length, decreased phosphate levels	yes	++

Supplementary Table 2. Correlations between transcripts from muscle biopsies and physiological parameters in postmenopausal women. Spearman correlations (ρ) were calculated for 18 women (except for muscle thickness, n=14). All parameters adjusted by age, height and fat mass percent, except for total body fat mass only adjusted by age and height. Only results for the Affymetrix probeset (in parenthesis) with the highest average signal value for each gene are shown.

Parameter	*RAI1* (8005267)		*SREBF1* (8013135)		*TOM1L2* (8013159)		*ATPAF2* (8013179)		*GID4* (8005305)	
	ρ	P	ρ	P	ρ	P	ρ	P	ρ	P
Total body BMD (g/cm2)	0.226	0.37	-0.463	0.053	-0.102	0.68	0.110	0.66	0.051	0.84
Femoral neck BMD (g/cm2)	0.288	0.28	**-0.773**	**6.8E-04**	-0.229	0.39	-0.282	0.29	-0.174	0.52
Total body lean mass (g)	-0.199	0.43	0.071	0.78	-0.094	0.71	-0.141	0.57	-0.017	0.95
Total body fat mass (g)	-0.223	0.37	-0.179	0.47	0.078	0.78	0.168	0.50	0.164	0.51
Muscle thickness (cm)	**-0.643**	**0.01**	-0.121	0.68	**0.622**	**0.02**	0.328	0.25	0.406	0.15

Supplementary Notes

Study Populations

The Generation R study

The Generation R study is a multiethnic prospective cohort study in which 9,778 pregnant women living in Rotterdam and with delivery date from April 2002 until January 2006 were enrolled. Details of study design and data collection can be found elsewhere[5]. The current study comprised 4,071 children, who had both GWAS and DXA-measurements. Total-body DXA measurements were performed using a GE Lunar iDXA densitometer on children accompanied by their mothers who were visiting a unique research centre at a mean age of 6.16 years (SD=0.39). All research aims and the specific measurements in the Generation R study have been approved by the Medical Ethical Committee of the Erasmus Medical Center, Rotterdam and written informed consent was provided by all parents or custodians. DNA samples were genotyped either with Illumina HumanHap 610 or Illumina HumanHap 660 bead chips. Duplicated samples or those observing excess of heterozygosity and gender mismatches were excluded from the dataset. SNPs with a minimum allele frequency (MAF) < 1%, call rate<98% or out of Hardy-Weinberg equilibrium ($P < 1x10^{-6}$) were removed from further analyses. All samples were imputed to the combined HapMap Phase II panel (build 36 release 22). Imputations were performed following a two-step procedure as proposed by the MACH/minimac suit [6]. Detailed information of the generation of the GWAS data can be found elsewhere[7]. For this study final filters of imputation quality (MACH R^2>=0.3) and allele frequency (MAF>=0.05) were applied.

Avon Longitudinal Study of Parents and their Children (ALSPAC)

The Avon Longitudinal Study of Parents and their Children (ALSPAC) is a longitudinal population-based birth cohort that recruited pregnant women residing in Avon, UK, with an

expected delivery date between 1st April 1991 and 31st December 1992. This cohort is described in detail on the website (http://www.alspac.bris.ac.uk) and elsewhere [8]. Please note that the study website contains details of all the data that is available through a fully searchable data dictionary (http://www.bris.ac.uk/alspac/researchers/data-access/data-dictionary/). Total-body DXA measurements and GWAS data were available for 5,251 unrelated children (mean age=9.9, SD=0.32 years) all of Northern-European descent. Ethical approval was obtained from the ALSPAC Law and Ethics committee and relevant local ethics committees, and all parents or custodians provided written informed consent. DNA samples were genotyped using the Illumina HumanHap550 bead chip. Individuals were excluded from further analysis on the basis of having incorrect gender assignments, minimal or excessive heterozygosity (< 0.320 and > 0.345 for the Sanger data and < 0.310 and > 0.330 for the LabCorp data), disproportionate levels of individual missingness (> 3%), evidence of cryptic relatedness (> 10% IBD) and being of non-European ancestry (as detected by a multidimensional scaling analysis seeded with HapMap Phase II individuals). EIGENSTRAT analysis revealed no additional obvious population stratification and genome-wide analyses with other phenotypes indicate low inflation of the test statistics (lambda) [9]. SNPs with a minor allele frequency of < 1% and call rate of < 95% were removed. Furthermore, only SNPs that passed an exact test of Hardy–Weinberg equilibrium ($P > 5\times10^{-7}$) were considered for analysis. All samples were imputed to the CEU panel of HapMap Phase II (build 36 release 22). Imputations were performed following a two-step procedure as proposed by the MACH/minimac suit [6]. For this study final filters of imputation quality (MACH R^2>=0.3) and allele frequency (MAF>=0.05) were applied.

Bone Mineral Density in Childhood Study (BMDCS)

The Bone Mineral Density in Childhood Study is an ongoing longitudinal study in which boys and girls aged 6-16 year old were recruited between 2002-2003, and whose DXA measurements are obtained annually at five clinical centers in the United States [10]. A total of 821 individuals (mean age =8.74, SD=1.91 years) with both DXA and GWAS data were included in the analyses described here. Participants underwent DXA scans in one of the five centers as part of the Bone Mineral Density in Childhood Study [11]. Total-body DXA scans were obtained using Hologic, Inc.

(Bedford, MA) bone densitometers. All samples were genotyped in the HumanOmniExpressExome-8v1. Samples with gender discrepancy, excess of heterozygosity, low genotype quality and sample replicates were excluded. SNPs with MAF < 1% and call rate of < 95% were removed. Furthermore, only SNPs which passed an exact test of Hardy–Weinberg equilibrium (P $>10^{-6}$) were considered for imputation. Imputations were performed following a two-step procedure; haplotype phasing was carried out using ShapeIT while imputation to the combined HapMap Phase II (build 36 release 22) reference was performed with IMPUTE2 [12]. Filters of imputation quality (info>=0.4) and allele frequency (MAF>=0.05) were applied.

The BMDCS principal investigators are:

Heidi J. Kalkwarf, PhD[1]; Joan M. Lappe, PhD[2]; Vicente Gilsanz, MD[3]; Sharon E. Oberfield, MD[4]; John A. Shepherd, PhD[5], Andrea Kelly, MD[6,7], Babette S. Zemel, PhD[6,8]

[1]Division of Gastroenterology, Hepatology and Nutrition, Cincinnati Children's Hospital Medical Center, Cincinnati. [2]Division of Endocrinology, Department of Medicine, Creighton University, Omaha. [3]Department of Radiology, Children's Hospital Los Angeles, Los Angeles. [4]Division of Pediatric Endocrinology, Diabetes, and Metabolism, Department of Pediatrics, Columbia University Medical Center, New York. [5]Department of Radiology, University of California San Francisco, San Francisco. [6]Department of Pediatrics, Perelman School of Medicine, University of Pennsylvania, Philadelphia. [7]Division of Endocrinology and Diabetes, The Children’s Hospital of Philadelphia, Philadelphia. [8]Division of Gastroenterology, Hepatology and Nutrition, The Children’s Hospital of Philadelphia, Philadelphia

Copenhagen Prospective Studies on Asthma (COPSAC) cohort

The Copenhagen Prospective Studies on Asthma in Childhood is a clinical study. All mothers had a history of a doctor’s diagnosis of asthma after 7 years of age. Newborns were enrolled in the first month of life, as previously described in detail [13]. The Ethics Committee for Copenhagen and the Danish Data Protection Agency approved this study. The current study comprised 273 children (mean age 6.89, SD=0.72 years), who had both GWAS data and DXA-measurements.

Total-body DXA measurements were assessed using a GE Lunar iDXA densitometer. Samples were genotyped using the Illumina HumanHap550 platform at the Children's Hospital of Philadelphia's Center for Applied Genomics. SNPs were excluded in basis of low call rate <95%, minor allele frequency (MAF) of <1% or Hardy-Weinberg equilibrium P value of $<1 \times 10^{-6}$. Imputation to the CEU panel of the HapMap Phase II (build 36 release 22) reference was performed in the MACH/minimac suit [6]. For this study final filters of imputation quality (MACH R^2>=0.3) and allele frequency (MAF>=0.05) were applied.

Description of TBLM/TBLH-BMD Associated Loci

Seven of the eight loci associated with TBLH-BMD and TBLM in this pediatric bivariate meta-analysis were previously described in GWAS of BMD at different skeletal sites. In the 1p36.12 locus, the bivariate signal arises from a region in linkage disequilibrium (LD) extending 264 Kb, with GWS variants located within *WNT4* and in close vicinity to the 5'region of *ZBTB40* (lead SNP rs6684375, P=$2.8x10^{-9}$). The low LD between the SNPs located at the opposite ends of the described region (r^2=0.005) are indicative of at least two independent association signals mapping to this locus[14]. GWS variants harboured in the 2q24.3 locus are in high LD and located in the vicinity of the 3'region of *CSRNP3* and *GALNT3* (lead SNP rs6726821, P=$2.8x10^{-8}$)[14,15]. The signal in the 4q22.1 locus maps to the vicinity of the 3'region of *MEPE* (lead SNP rs11733405, P=$3.3x10^{-8}$)[14,15]. The peak in the 7q31.31 locus spans for 307 Kb in an LD region harbouring *CPED1*, *WNT16,* and *FAM3C.* The LD structure of this region is compatible with the presence of different signals underlying the captured association (lead SNP rs917727, P=$3.1x10^{-20}$)[15,16]. SNPs in the 11q13.2 locus are in high LD and spread throughout the *PPP6R3* gene region. Moreover, they are in moderate correlation with markers mapping to the 3'region of *LRP5* (lead SNP rs12271290, P=$4.4x10^{-9}$), a well-established BMD and fracture locus[14,17]. Variants underlying the signal in the 13q14.11 locus are in high LD and located in *TNFSF11* or its vicinity (lead SNP rs9525638, P=$1.4x10^{-8}$)[14]. The signal on the 14q2.12 locus arises from variants mapping to *RIN3* (lead SNP rs754388, P=$3.3x10^{-8}$), previously associated with TBLH-BMD in children[15] and a determinant of Paget's disease[18].

Supplementary Methods

Genetic Relatedness Matrix Generation for the Generation R Study

As data from Generation R Study comes from and admixed population and the estimation of the relationship coefficient from GCTA under this scenario suffer a bias. One important assumption of this method is that the sampling population is an homogeneous population, the equations defining the relatedness coefficient are largely dependent on the population allele frequency; as in admixed population the population allele frequency will not necessarily represent the allele frequency of any subpopulation problems emerge: a systematic inflation on the estimated degree of relatedness, particularly in unrelated pair of individuals who have either the same ancestry or similar admixed ancestry [19]. Recently, Thornton et al. created a software REAP (Relatedness Estimation in Admixed Populations), which is able to estimate accurately admixed population relatedness estimates by using individual-specific allele frequencies that are calculated by conditioning on estimated individual genome-wide ancestry [19]. This issue becomes of importance when high sensitivity is required (i.e., for the assessment of distant relatedness and/or fine pedigree structure), as it is the case of heritability estimates. Hence, we have modified the genetic relationship matrix (GRM) by using the REAP estimator as follows.

The GRM coefficient from the GCTA methodology as described in [20] is

$$A_{jk} = \frac{1}{N}\sum_{i=1}^{n}\frac{(x_{ij}-2p_i)(x_{ik}-2p_i)}{2p_i(1-p_i)}$$

Where A_{jk} is the relatedness coefficient for individuals *j* and *k*. N the number of SNPs under study. x_i the genotype of individual *j* or *k* at marker I (e.g., 0,1,2) and p_i the population minor allele frequency of marker *i*.

REAP condition the population frequency for each individual p_m a linear combination of the subpopulation allele frequencies based on individual's ancestry [19] and thus,

$$A_{jk} = \frac{1}{N} \sum_{i=1}^{n} \frac{(x_{ij} - 2p_{mj})(x_{ik} - 2p_{mk})}{\sqrt{p_{mj}(1 - p_{mj})}\sqrt{p_{mk}(1 - p_{mk})}}$$

As originally REAP by default produce kinship coefficients ϕ_{jk} the coefficients reported should be multiplied by 2 as shown in [19].

$$\phi_{jk} = \frac{1}{2} A_{jk}$$

Supplementary References

1. Westra, H.J. *et al.* Systematic identification of trans eQTLs as putative drivers of known disease associations. *Nature Genetics* **45**, 1238-U195 (2013).
2. Consortium, G.T. Human genomics. The Genotype-Tissue Expression (GTEx) pilot analysis: multitissue gene regulation in humans. *Science* **348**, 648-60 (2015).
3. Bi, W. *et al.* Inactivation of Rai1 in mice recapitulates phenotypes observed in chromosome engineered mouse models for Smith-Magenis syndrome. *Hum Mol Genet* **14**, 983-95 (2005).
4. Girirajan, S. *et al.* Tom1l2 hypomorphic mice exhibit increased incidence of infections and tumors and abnormal immunologic response. *Mamm Genome* **19**, 246-62 (2008).
5. Kooijman, M.N. *et al.* The Generation R Study: design and cohort update 2017. *Eur J Epidemiol* **31**, 1243-1264 (2016).
6. Li, Y., Willer, C.J., Ding, J., Scheet, P. & Abecasis, G.R. MaCH: using sequence and genotype data to estimate haplotypes and unobserved genotypes. *Genet Epidemiol* **34**, 816-34 (2010).
7. Medina-Gomez, C. *et al.* Challenges in conducting genome-wide association studies in highly admixed multi-ethnic populations: the Generation R Study. *Eur J Epidemiol* **30**, 317-30 (2015).
8. Boyd, A. *et al.* Cohort Profile: The 'Children of the 90s'-the index offspring of the Avon Longitudinal Study of Parents and Children. *International Journal of Epidemiology* **42**, 111-127 (2013).
9. Price, A.L. *et al.* Principal components analysis corrects for stratification in genome-wide association studies. *Nat Genet* **38**, 904-9 (2006).
10. Kalkwarf, H.J. *et al.* The bone mineral density in childhood study: bone mineral content and density according to age, sex, and race. *J Clin Endocrinol Metab* **92**, 2087-99 (2007).
11. Zemel, B.S. *et al.* Revised reference curves for bone mineral content and areal bone mineral density according to age and sex for black and non-black children: results of the bone mineral density in childhood study. *J Clin Endocrinol Metab* **96**, 3160-9 (2011).
12. Howie, B.N., Donnelly, P. & Marchini, J. A flexible and accurate genotype imputation method for the next generation of genome-wide association studies. *PLoS Genet* **5**, e1000529 (2009).
13. Bisgaard, H. The Copenhagen Prospective Study on Asthma in Childhood (COPSAC): design, rationale, and baseline data from a longitudinal birth cohort study. *Ann Allergy Asthma Immunol* **93**, 381-9 (2004).
14. Estrada, K. *et al.* Genome-wide meta-analysis identifies 56 bone mineral density loci and reveals 14 loci associated with risk of fracture. *Nat Genet* **44**, 491-501 (2012).
15. Kemp, J.P. *et al.* Phenotypic dissection of bone mineral density reveals skeletal site specificity and facilitates the identification of novel loci in the genetic regulation of bone mass attainment. *PLoS Genet* **10**, e1004423 (2014).
16. Medina-Gomez, C. *et al.* Meta-analysis of genome-wide scans for total body BMD in children and adults reveals allelic heterogeneity and age-specific effects at the WNT16 locus. *PLoS Genet* **8**, e1002718 (2012).
17. Richards, J.B. *et al.* Bone mineral density, osteoporosis, and osteoporotic fractures: a genome-wide association study. *Lancet* **371**, 1505-12 (2008).
18. Albagha, O.M. *et al.* Genome-wide association identifies three new susceptibility loci for Paget's disease of bone. *Nat Genet* **43**, 685-9 (2011).
19. Thornton, T. *et al.* Estimating kinship in admixed populations. *Am J Hum Genet* **91**, 122-38 (2012).

20. Yang, J., Lee, S.H., Goddard, M.E. & Visscher, P.M. GCTA: a tool for genome-wide complex trait analysis. *Am J Hum Genet* **88**, 76-82 (2011).
